# Volume-amplified magnetic bioassay integrated with microfluidic sample handling and high-*T_c_* SQUID magnetic readout

**DOI:** 10.1063/1.4999713

**Published:** 2017-12-29

**Authors:** Sobhan Sepehri, Emil Eriksson, Alexei Kalaboukhov, Teresa Zardán Gómez de la Torre, Kiryl Kustanovich, Aldo Jesorka, Justin F. Schneiderman, Jakob Blomgren, Christer Johansson, Maria Strømme, Dag Winkler

**Affiliations:** 1 Department of Microtechnology and Nanoscience–MC2, Chalmers University of Technology, Göteborg 412 96, Sweden; 2 RISE Acreo AB, SE-411 33 Göteborg, Sweden; 3 Department of Engineering Sciences, Uppsala University, The Ångström Laboratory, Box 534, SE-751 21 Uppsala, Sweden; 4 Department of Chemistry and Chemical Engineering, Chalmers University of Technology, Göteborg 412 96, Sweden; 5 MedTech West and the Institute of Neuroscience and Physiology, University of Gothenburg, SE-40530 Göteborg, Sweden

## Abstract

A bioassay based on a high-*T_c_* superconducting quantum interference device (SQUID) reading out functionalized magnetic nanoparticles (fMNPs) in a prototype microfluidic platform is presented. The target molecule recognition is based on volume amplification using padlock-probe-ligation followed by rolling circle amplification (RCA). The MNPs are functionalized with single-stranded oligonucleotides, which give a specific binding of the MNPs to the large RCA coil product, resulting in a large change in the amplitude of the imaginary part of the ac magnetic susceptibility. The RCA products from amplification of synthetic *Vibrio cholera* target DNA were investigated using our SQUID ac susceptibility system in microfluidic channel with an equivalent sample volume of 3 *μ*l. From extrapolation of the linear dependence of the SQUID signal versus concentration of the RCA coils, it is found that the projected limit of detection for our system is about 1.0 × 10^5^ RCA coils (0.2 × 10^−18^ mol), which is equivalent to 66 fM in the 3 *μ*l sample volume. This ultra-high magnetic sensitivity and integration with microfluidic sample handling are critical steps towards magnetic bioassays for rapid detection of DNA and RNA targets at the point of care.

## I. INTRODUCTION

Bioassays were developed decades ago as analytical techniques with widespread applications such as clinical chemistry and drug analysis. They use different mechanisms to translate the bimolecular interactions that measure the presence or concentration of biological or chemical molecules into a physical signal. The golden standard immunoassay, enzyme-linked immunosorbent assay (ELISA), is based on either a radioactive or fluorescent tag or an enzyme label (Lequin, 2005). Developments in the synthesis and coatings of magnetic nanoparticles (MNPs) (Lu *et al.*, 2007) have given them a biological recognition function to be used for antigen-antibody binding or ligand-receptor binding. Due to the large surface to volume ratio of nanoparticles, they provide mobile substrates suspended in the fluid for binding to target analyte, eliminating the need for washing steps and immobilization. In addition to providing a mobile binding surface, MNPs can also be used as detection labels (Tekin and Gijs, 2013) due to their magnetic properties. Exploiting the magnetic properties of the nanoparticles as the physical signal enables real time detection even in opaque liquids such as blood.

Various magnetic readout schemes based on different sensing principles have been developed for biomolecule detection using MNP labels (Schrittwieser *et al.*, 2016). One approach is to determine the change in hydrodynamic volume of the MNPs by measuring the change in Brownian relaxation dynamics. Proposed theoretically by Connolly and St Pierre (Connolly and St Pierre, 2001) and shown experimentally for prostate-specific antigen (PSA) and Brucella antibodies (Astalan *et al.*, 2004; Fornara *et al.*, 2008), the change in Brownian relaxation time due to binding to analytes was determined by measuring complex frequency dependent magnetic susceptibility. Examples of sensors that utilize this dynamic mechanism are high-*T*_c_ superconducting quantum interference devices (SQUIDs) (Enpuku *et al.*, 2017; Yang *et al.*, 2013), the differential induction coil system DynoMag (RISE Acreo, Sweden) (Astalan *et al.*, 2004; Zardán Gómez de la Torre *et al.*, 2011), and opto-magnetic sensors (Donolato *et al.*, 2015). These sensors exploit the three dimensions of sample volume for analyte and probe binding and measure the signal generated from the whole sample volume. Therefore, they are more favorable compared to Hall effect sensors (Sandhu *et al.*, 2004) and giant magnetoresistive sensors (Wang *et al.*, 2014) which rely on the diffusion of MNP labels toward the two dimensional sensor surface.

We have previously developed our high-*T*_c_ SQUID based magnetic ac susceptibility immunoassay (Öisjöen *et al.*, 2009). We were able to detect PSA10 molecules using antigen-antibody binding reaction with a concentration of 0.7 nM (nanomole/l) within a 2 *μ*l droplet (Öisjöen *et al.*, 2010). The reaction takes place on the surface of functionalized magnetic nanoparticles (fMNPs) and results in a relatively small change in the effective hydrodynamic volume of MNPs and, in turn, a small shift in relaxation frequency. Recently, rolling circle amplification (RCA) (Banér *et al.*, 1998), has been implemented to increase the sensitivity of MNP based bioassays by at least a factor of 1000 (Zardán Gómez de la Torre *et al.*, 2011) compared to studies that look into the Brownian frequency shift. The target molecule recognition is based on padlock-probe-ligation followed by RCA for volume amplification. The magnetic nanoparticles are bound to the large RCA coils (with diameter about 1 *μ*m), resulting in a significant shift in the imaginary component of ac susceptibility which corresponds to the Brownian relaxation frequency of the MNPs. The amount of target molecules can therefore be obtained from the number of MNPs that have not bound to the RCA coils after hybridization as a change in the amplitude of the Brownian relaxation frequency peak of the fMNPs. The RCA/fMNP bioassay has been measured using the DynoMag and the opto-magnetic system and shows significant improvement in sensitivity. RCA coils produced from *Vibrio cholera* target DNA were investigated in the DynoMag system using 100 nm fMNPs, and the estimated theoretical limit of detection (LOD) in 200 *μ*l sample volume was 55 fM (femtomole/l) (Ahrentorp *et al.*, 2016). In the opto-magnetic sensor, a LOD of 780 fM RCA coils produced from the *Salmonella* DNA target was achieved in a sample volume of 65 *μ*l (Tian *et al.*, 2016).

In this work, we used the RCA volume amplified magnetic bioassay with our high-*T*_c_ SQUID gradiometer as the magnetic readout. We also take advantage of microfluidic chips for sample handling for accurate measurements of small volumes of analyte. The synthetic DNA target molecules from *V. cholera* were recognized and amplified to large coils using padlock probe hybridization and RCA. Measuring the ac susceptibility to determine the change in the Brownian relaxation dynamics of the MNPs due to hybridization with the RCA coils with various concentrations, we estimated the LOD for our system to be about 1.0 × 10^5^ RCA coils (0.2 × 10^−18^ mol), which is equivalent to 66 fM in the 3 *μ*l sample volume. The method and instruments that are adopted and presented in this paper for synthetic *V. cholera* as the target bioanalyte are generic and could be used for other DNA or RNA viruses. We discuss future implementation of all steps of the bioassay on a disposable lab-on-chip system for rapid, easy, and cheap point-of-care diagnosis.

## II. EXPERIMENTAL RESULTS

### A. Free magnetic nanoparticles

**FIG. 1.**
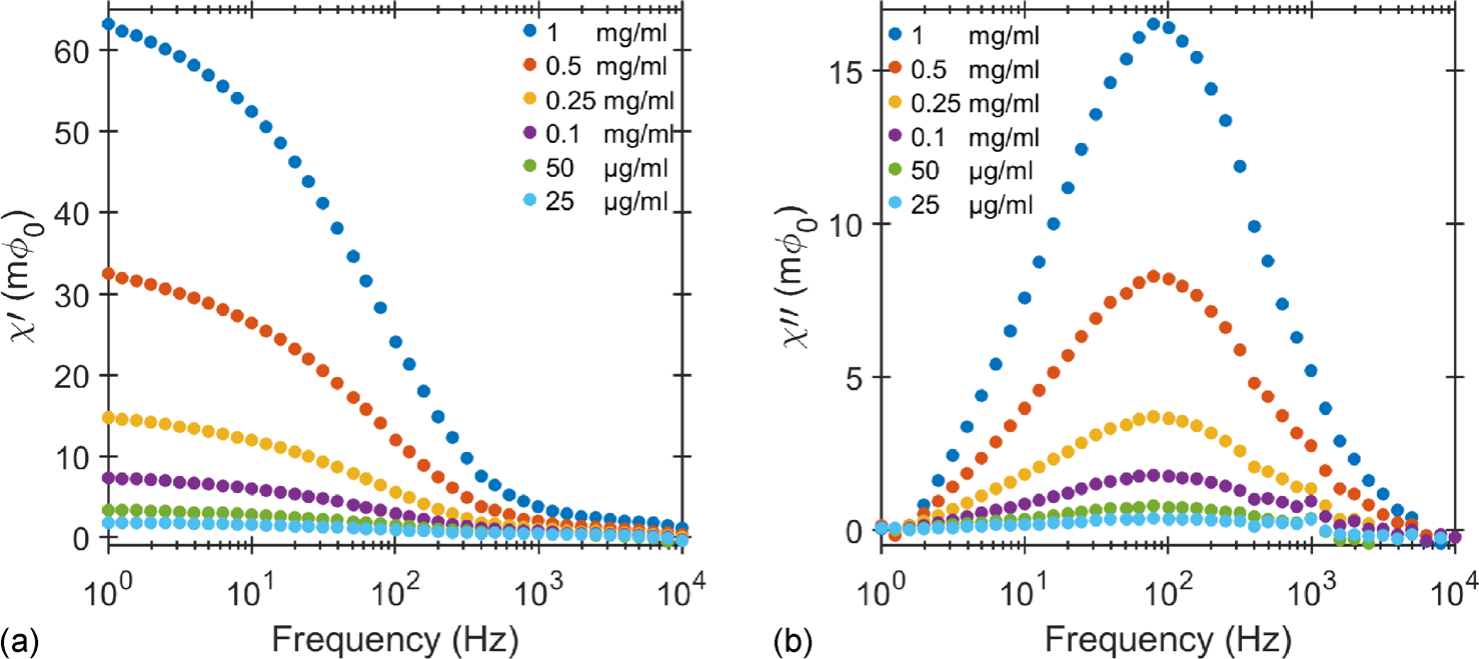
(a) In-phase and (b) out-of-phase components of the SQUID signal versus the excitation field frequency for different concentrations of streptavidin coated MNPs at a field strength of 40 *μ*T. Given the $1\;V/\Phi_{0}$ transfer function for the SQUID, the voltage output of the SQUID was converted into flux quantum $\Phi_{0}=2\times 10^{-15}\;\text{Wb}$. The in-phase and out-of-phase components are related to the real and imaginary components of ac susceptibility, respectively. The maximum of imaginary ac susceptibility frequency is at 80 Hz.

**FIG. 2.**
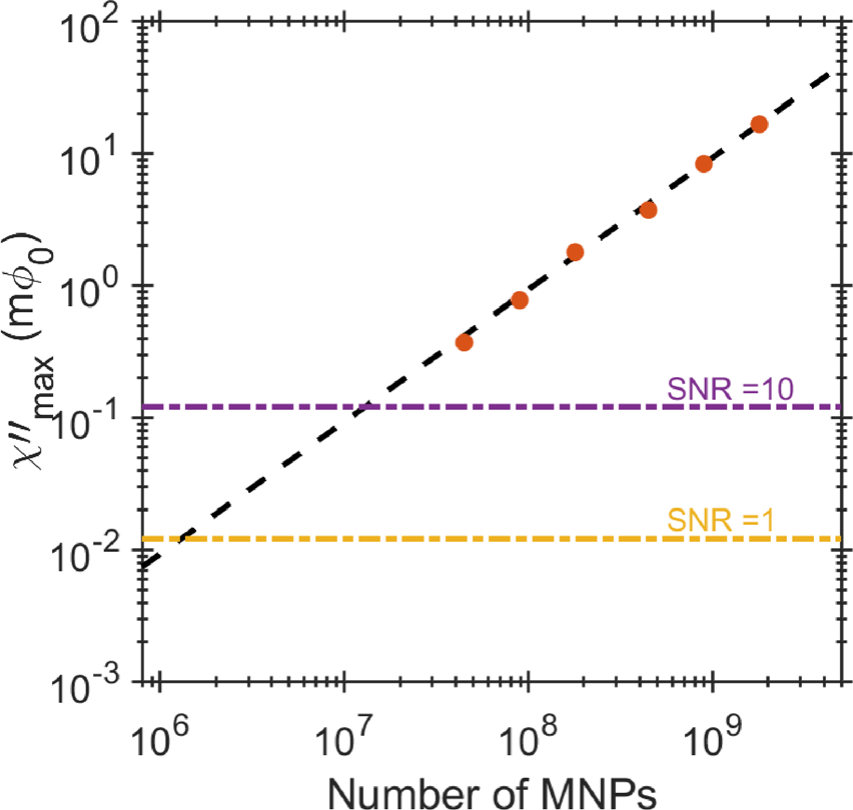
The peak of the imaginary part of the complex susceptibility is plotted against the number of MNPs. Linear extrapolation of the peak amplitude dependency to the number of MNPs gives the sensitivity of 1.5 × 10^6^ MNPs at SNR = 1.

The real and imaginary components of complex susceptibility versus the excitation field frequency for various concentrations of non-functionalized MNPs suspended in phosphate-buffered saline (PBS) are shown in Figs. 1(a) and 1(b). The imaginary part of the susceptibility peaks at the Brownian relaxation frequency at around 80 Hz, which corresponds to the median particle size of 100 nm according to Eq. (1). In order to accurately find the frequency of the imaginary ac susceptibility peak, we have used the empirical Cole-Cole formula (Cole and Cole, 1941). The amplitude of both real and imaginary parts of the susceptibility decreases with decreasing concentration of MNPs, but there is no frequency shift in the peak position of the imaginary part. Figure 2 shows the linear relation between the amplitude of the peak and the number of MNPs estimated from the known number of particles per unit volume, see Sec. V D. The vertical axis corresponds to the SQUID output signal in the units of the magnetic flux quantum (Φ_0_ = 2×10^−15^ Wb). The extrapolation of this linear dependence to the measured SQUID noise of $1.2\times {10^{-5}\Phi }_{0}/\sqrt{\text{Hz}\;}\;$ allows estimation of the LOD of our system to be 1.5 × 10^6^ MNPs $/\sqrt{\text{Hz}\;}$ or 2.9 × 10^−10^ $\text{emu}/\sqrt{\text{Hz}\;}$ in magnetic moment.

### B. Measurement of RCA coils

**FIG. 3.**
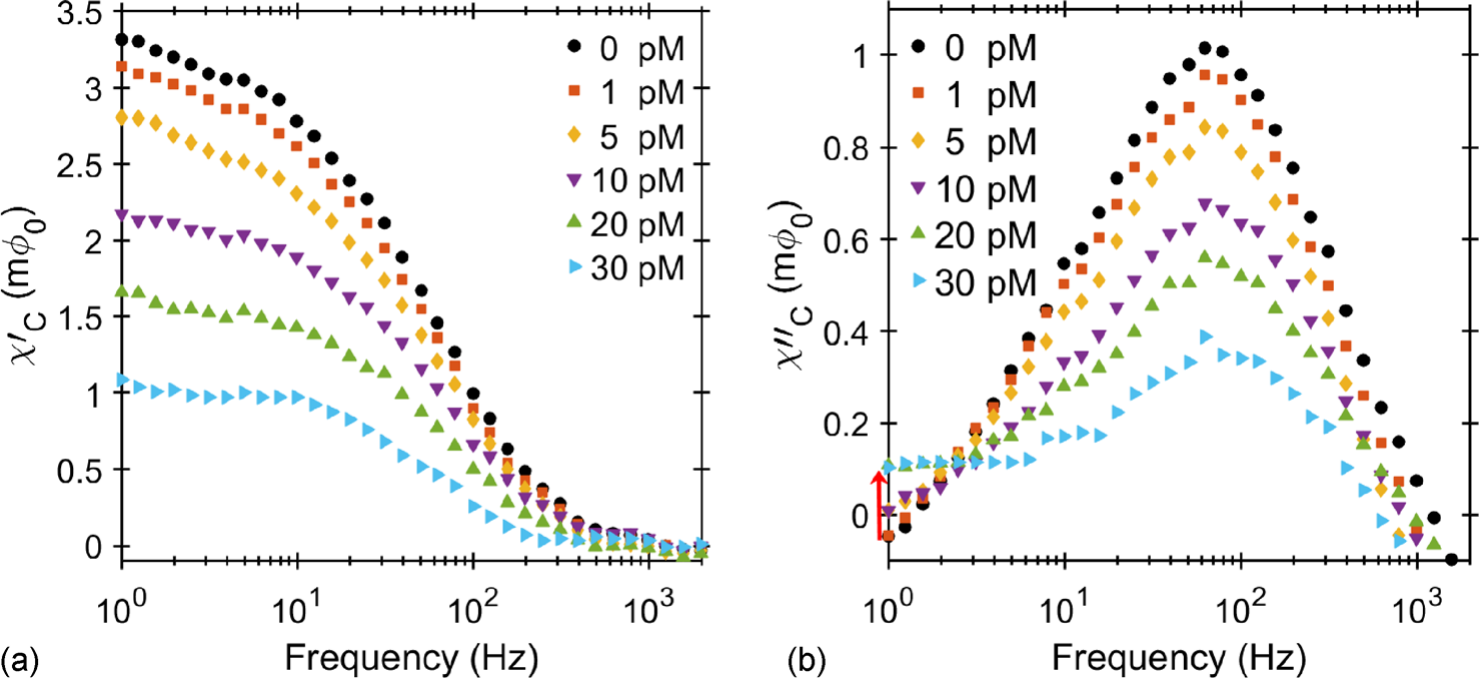
(a) In-phase and (b) out-of-phase components of the SQUID signal ($1\;V/\Phi_{0}$ transfer function) versus the excitation frequency for different concentrations of RCA coils ranging from 0 (NC) to 30 pM in a total volume of 3 *μ*l. The in-phase and out-of-phase components are related to real and imaginary ac susceptibilities, respectively. The drop in the amplitude of both real and imaginary components of the susceptibility indicates the increase in the number of RCA coils and fewer numbers of free/unbound MNPs in the solution. The red arrow shows a rise in the low frequency tail of the response with the increasing RCA concentration (H = 40 *μ*T).

The real and imaginary components of the ac susceptibility signal from samples with various concentrations of RCA coils, from 0 to 30 pM (picomole/l), versus the excitation field frequency in the range of 1 to 3000 Hz are shown in Figs. 3(a) and 3(b). The zero concentration (0 pM) is the negative control (NC) sample that contains only fMNPs with a mass concentration of 50 *μ*g/ml. From the Cole-Cole fit of the imaginary ac susceptibility component for the NC sample, it is estimated that the imaginary susceptibility is maximum at 60 Hz. The frequency of the peak (Brownian relaxation frequency) is lower compared to that of non-functionalized MNPs due to the presence of oligonucleotides on the surface of the MNPs which increases the hydrodynamic volume and in turn decreases the Brownian relaxation frequency (from 80 Hz to 60 Hz).

Both the real and imaginary ac susceptibility components decrease as the concentration of RCA coils increases, see Figs. 3(a) and 3(b), respectively. The continuous decrease in the 60 Hz peak amplitude of the imaginary component with the increasing RCA concentration is due to the decrease in the number of free fMNPs in solution, while more of the fMNPs are hybridized (and hence immobilized) to the RCA coils. When the fMNPs are bound to the very large RCA coil, their effective hydrodynamic volume increases and the Brownian relaxation frequency falls well below 10 Hz (the Brownian relaxation frequency of the 1 *μ*m RCA coils is in the range of 0.4 Hz). The signature of this low frequency peak is seen as a rise in the imaginary magnetic susceptibility signal below 10 Hz, indicated with a red arrow in Fig. 3(b). The amplitude of this low frequency peak becomes more pronounced for higher concentrations of RCA coils because of the greater ratio of the RCA coil bound fMNPs to unbound fMNPs. The fewer unbound fMNPs explain the decrease in the amplitude of the real component at 1 Hz. It is also interesting to mention that the frequency at which the imaginary ac susceptibility maximizes increases with the increasing RCA concentration. This shows that the larger MNPs in the sample distribution take precedence over smaller ones in hybridizing with RCA coils (Ahrentorp *et al.*, 2016).

**FIG. 4.**
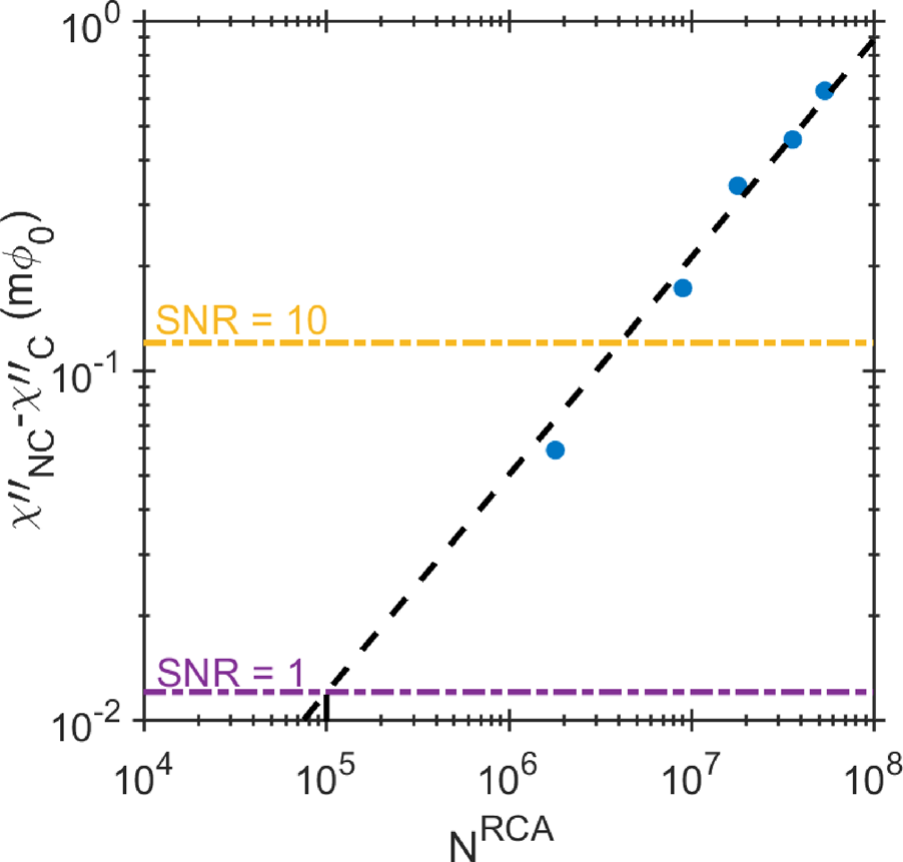
The extinction signal ($\chi_{NC}^{\prime \prime }-\chi_{C}^{\prime \prime }$) for different concentrations of the RCA coils at the peak frequency of 60 Hz is plotted versus the number of RCA coils in the corresponding concentration. The linear extrapolation gives 1.0 × 10^5^ LOD to RCA coils in a 3 *μ*l sample volume at SNR = 1.

To estimate the LOD for RCA coils, we obtained the amplitudes of the imaginary component of the ac susceptibility at 60 Hz for every RCA concentration. The extinction is a difference between these peak amplitudes and the NC sample (0 pM concentration) peak amplitude, $\chi_{NC}^{\prime \prime }-\chi_{C}^{\prime \prime },$ and therefore determines the number of MNPs that were bound to the RCA coils, see Fig. 4. Extrapolating the linear dependence of extinction to the noise of our sensor corresponds to a theoretical detection limit of about 1.0 × 10^5^ RCA coils, which is equivalent to 66 fM of target analyte in the 3 *μ*l sample volume. We can also estimate the average number of MNPs that are conjugated to each individual RCA coil. Since we have the same concentration of fMNPs (50 *μ*g/ml) in each of the RCA concentration samples, the extinction of their ac susceptibility signal magnitude compared to the NC, the black dotted line in Figs. 3(a) and 3(b), corresponds to the number of fMNPs bound to RCA coils. Using the slope, *S*, of the linear dependence of the signal on the number of MNPs, Fig. 2, we can estimate the remaining number of unbound fMNPs, $N_{C}^{\text{unbnd}},$ and the number of fMNPs bound to RCA coils, $N_{C}^{bnd}$

$$N_{C}^{\text{unbnd}}=\frac{\chi_{C}^{\prime \prime }}{S},$$

$$N_{C}^{bnd}=\;N_{NC}-N_{C}^{unbnd},$$

where $\chi_{C}^{\prime \prime }$ is the peak imaginary ac susceptibility signal for the RCA concentration of C sample and $N_{NC}$ is the number of particles in the NC sample. Dividing the number of bound MNPs by the number of RCA coils for each RCA concentration gives the average number of MNPs per RCA coil, $g_{MNP}$

$$g_{MNP}=\frac{N_{C}^{bnd}}{N_{C}^{RCA}}=\frac{1}{N_{C}^{RCA}\times S\;}\left(\chi_{NC}^{\prime \prime }-\chi_{C}^{\prime \prime }\right),$$

where $\chi_{NC}^{\prime \prime }$ is the peak imaginary ac susceptibility for the NC sample and $N_{C}^{RCA}$ is the number of RCA coils in the sample with the RCA coil concentration of *C*. Estimated $g_{MNP}$ from the lowest to highest concentrations of RCA coils differs from 3.6 to 1.3 MNP per coil. We obtain on average 2 MNPs per RCA coil, which is a close estimation to the evaluated mean value of 3 MNP per RCA coil using the DynoMag system (Ahrentorp *et al.*, 2016). Transmission electron microscopy (TEM) analysis of RCA coils conjugated with 130 nm MNPs also shows that the average number of MNPs per coil is around 2 (Akhtar *et al.*, 2010).

## III. DISCUSSION

**FIG. 5.**
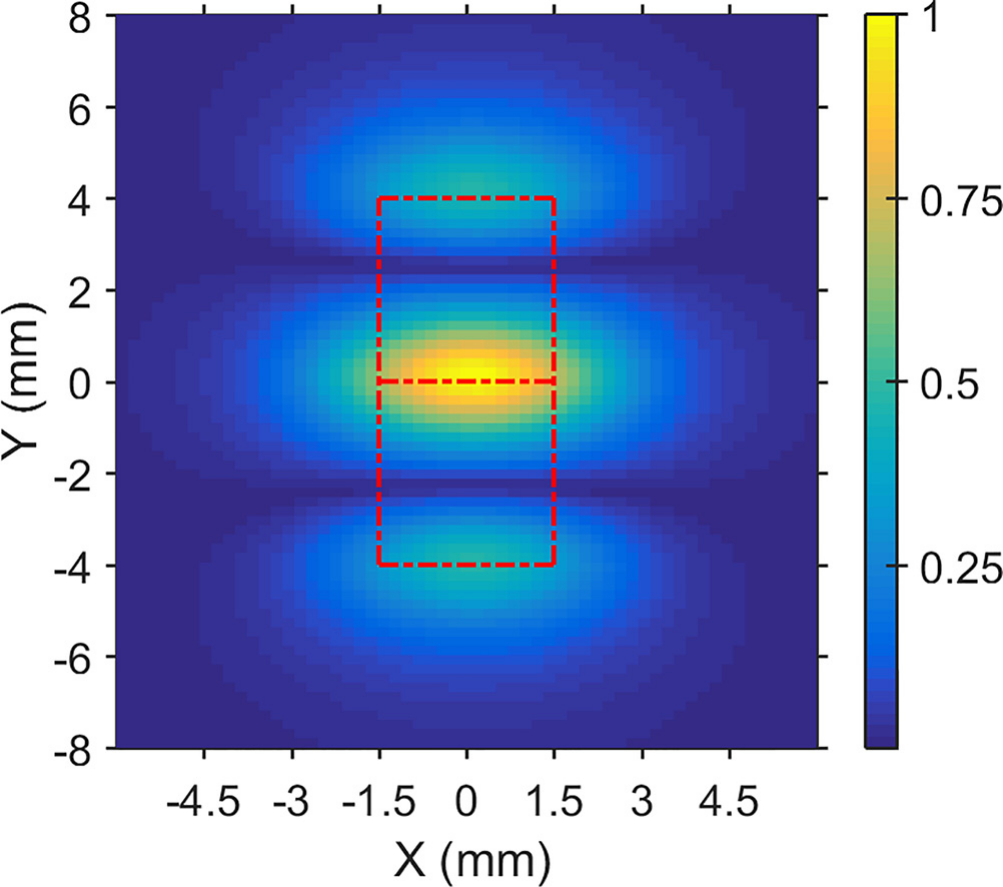
Simulation of absolute magnetic flux distribution from a colloidal sample with the 3 *μ*l volume size versus the XY coordinate. The bottom of the sample is placed in plane and at a constant 1.1 mm distance away from the gradiometer pick-up loops. The MNPs are randomly distributed in XY and Z directions of the 3 mm × 1 mm × 1 mm microfluidic channel. The center of the colloidal sample then sweeps the XY plane to get the flux distribution. The dashed lines indicate the edges of the gradiometer, and the color-coding represents the magnetic flux normalized to the maximum absolute flux threading the two loops of the gradiometer. The magnetic excitation field is in the y-direction, parallel to the baseline of the gradiometer.

The biosensing sensitivity of our magnetic bioassay is determined by the sensitivity of the magnetic readout and the number of MNPs conjugated per single RCA coil molecule. In this work, the sensitivity of our SQUID-based ac susceptibility system to magnetic materials has significantly improved from the previous result (Öisjöen *et al.*, 2010). The improvement in sensitivity is mainly due to utilizing the microfluidic channel for sample handling. The microfluidic channel allowed us to control the sample geometry and position, which realizes a better coupling to the pick-up loops of the SQUID. In a previous work using electrowetting-on-dielectrics (EWOD) for sample handling and manipulation, we have demonstrated the advantages of using small sample volumes compared to large volume sizes. The picked-up signal from droplets as small as 2 *μ*l was 2.5 times larger than the bulk samples of 30 *μ*l in volume (Schaller *et al.*, 2009). We performed modeling of the suspended MNPs as point-like magnetic dipoles randomly distributed in the specific volume and geometry of the microchannel and estimated the dependency of the magnetic flux as a function of the location of the microchannel with respect to the pick-up loop of the gradiometer, see Fig. 5. The results show that for the excitation field parallel to the base line of the gradiometer and for our 1 × 1 × 3 mm^3^ microchannel, the maximum coupling to the gradiometer loops occurs when the channel is placed in the middle of the baseline.

The equivalent magnetic sensitivity of our system is also about 20 times better than the induction coil DynoMag system (Ahrentorp *et al.*, 2016). However, we use rather low excitation field amplitude as compared to the induction coil system. Increasing the excitation field by one order of magnitude would proportionally increase the sensitivity of our system. Since the signal in the induction coil system depends linearly on frequency, it is important to emphasize that the sensitivity of our system is much better at low frequencies as compared to induction coil readout. This allows the measurements of ac susceptibility signals down to 1 Hz.

The high magnetic sensitivity results in a high LOD of RCA products of about 0.2 × 10^−18 ^mol as compared to 11 × 10^−18^ mol for the induction coil system. However, the molar concentration sensitivity of 66 fM is lower as compared to the DynoMag readout, 55 fM, because we use a smaller sample volume (3 *μ*l vs. 200 *μ*l). The main limiting factor at present is the low number of fMNPs hybridized to each RCA coil, on average only about 2–3 fMNPs per coil. The difference in the number of fMNPs per RCA coil is due to slightly different incubation volumes used during the hybridization process. This results in lower concentrations of fMNPs for the higher concentration of RCA coils. Since the binding kinetics mainly depends on the concentration of fMNPs (in the saturation regime), the number of fMNPs per RCA coil decreases with increasing RCA coil concentration. This number may be increased as there are many more binding sites available on each RCA coil. An additional amplification step can also be introduced to increase the number of RCA coils during the sample preparation step. Circle to circle amplification is an isothermal method with low amplification bias that monomerizes each RCA coil, circularizes the monomers again, and amplifies the circular molecules through RCA in order to generate a new set of RCA products (Kuhnemund *et al.*, 2014). Increasing the number of amplification steps, however, increases the time needed to run the entire bioassay.

The overall ultimate sensitivity of our ac susceptibility system is comparable with state-of-the-art ELISA bioassay (Wilson, 2013). However, our magnetic assay is much faster (2 h) and does not require multiple washing steps. Moreover, there is a very low background signal since the detection signal is the imaginary component of the ac susceptibility and comes only from the MNP response. It is therefore very promising for implementation in future point-of-care diagnostic systems. One of the drawbacks is the need for low temperature to operate our high-*T*_c_ SQUIDs. Recently, we have demonstrated the successful operation and noise measurements of a high-*T*_c_ SQUID utilizing a commercial two stage micro-electromechanical system (MEMS) based Joule-Thomson micro-cooler, CryoLab from Kryoz Technologies BV (Kalabukhov *et al.*, 2016). The micro-cooler offers a long operation time, simple usage, and temperature stability and adjustment. It can also be integrated with the disposable lab-on-a-chip microfluidic sample handler.

## IV. CONCLUSION

We have presented a sensitive magnetic bioassay based on a high-*T*_c_ SQUID gradiometer sensor which implements RCA as an amplification technique for the detection of DNA target molecules. The integration of microfluidic channels with the ac susceptibility measurement setup improved the magnetic sensitivity and resulted in an LOD to target analyte of 0.2 × 10^−18^ mol that is comparable to the state-of-the-art ELISA assays. The ultra-high sensitivity combined with short turn-around-time is promising for applications in the future point of care diagnostic system, where all steps of the assay are implemented on a disposable lab-on-a-chip. The assay is not limited to a single target molecule and can be adopted for various DNA and RNA targets.

## V. METHODS

### A. SQUID-based gradiometer sensor

We use a first order planar high-*T*_c_ dc SQUID gradiometer. The SQUID is fabricated from a single layer superconducting YBa_2_Cu_3_O_7_ film grown on a 10 mm × 10 mm SrTiO_3_ bicrystal substrate with a symmetric 24° c-axis tilt angle. The details of the SQUID fabrication and characterizations were presented in Öisjöen *et al.* (2008). The advantage of the gradiometer configuration is the elimination of the homogeneous magnetic field from the environmental sources. Our SQUID has an equivalent magnetic flux noise of $1.2\times {10^{-5}\Phi }_{0}/\sqrt{\text{Hz}\;}$ corresponding to an equivalent magnetic field gradient noise of ${2.1\times 10}^{-12}\;T/\text{cm}\sqrt{\text{Hz}\;}$ for our 3 mm baseline.

### B. Microfluidic channel

**FIG. 6.**
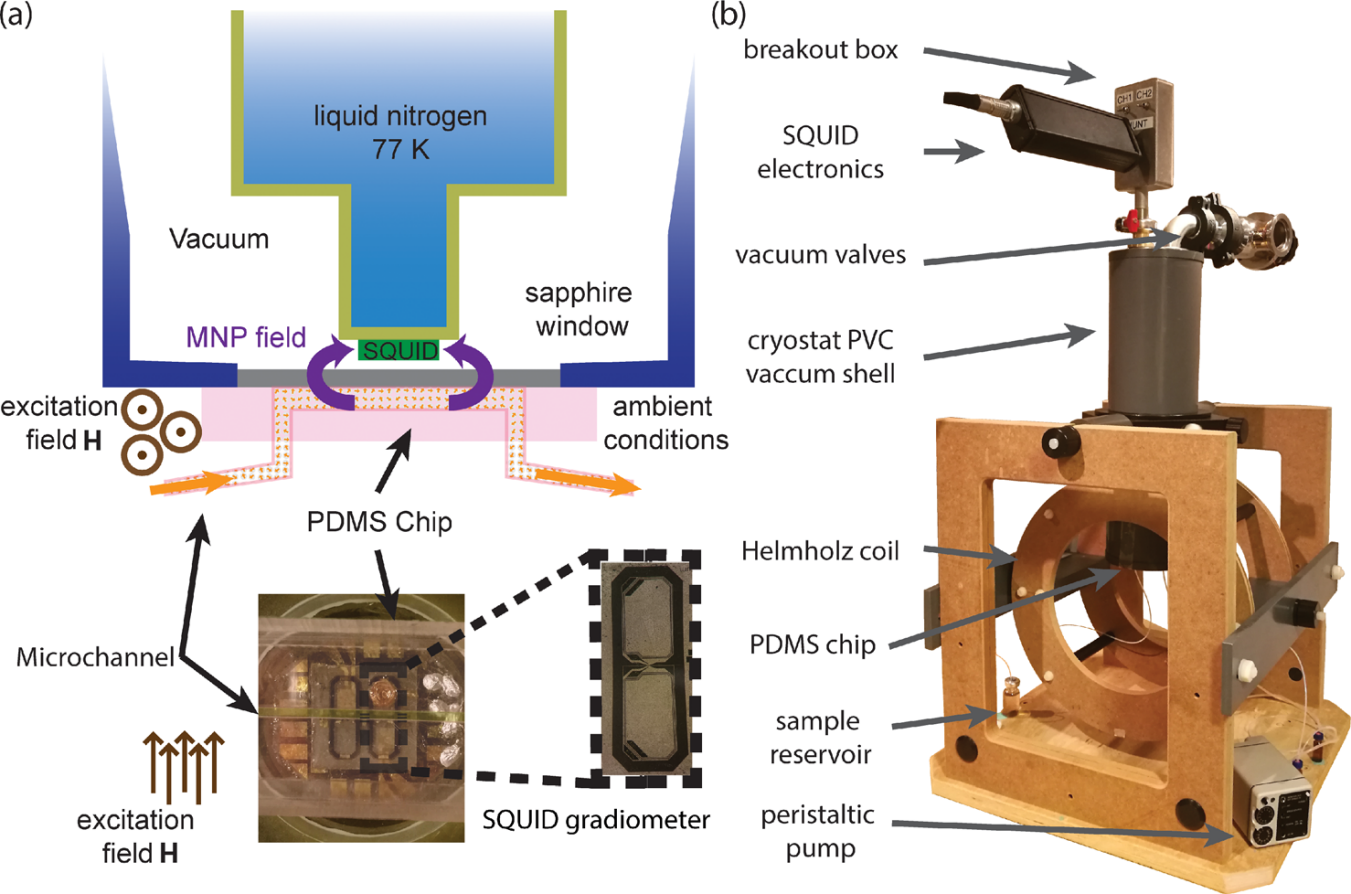
(a) Illustration of the microchannel placed on the sapphire window of the SQUID-based ac susceptibility setup and a photograph of the top view of the microchannel aligned on the sensitive part of the SQUID gradiometer. The dimensions of the channel are 1 × 1 ×3 mm^3^ with the effective volume of 3 *μ*l. (b) The in-house built cryostat sitting on an alignment frame with the Helmholtz coil to provide the excitation field.

A schematic illustration and a top view image of the microchannel placed above the SQUID sensor are given in Fig. 6(a). Microfluidic channels were fabricated using a silicone based organic polymer (polydimethylsiloxane, PDMS). The channel is 1 mm wide and 1 mm deep with a 100 *μ*m membrane that seals the channel on one side. It is manually aligned on the sapphire window in plane with the SQUID gradiometer for measurements. The MNPs are transported from a reservoir bottle using a peristaltic pump and then out from the outlet to a waste bottle. We use water to flush the channel to wash any residues inside, and therefore, there is no need for exchanging the microfluidic channel for repeated measurements. The microchannel allows fixed geometry and precise positioning of the sample. The thin membrane minimizes the distance between the sensor and the MNPs, which in turn increases the coupling between the MNPs and the pick-up loops of the sensor.

### C. Ac susceptibility measurement setup

Figure 6(b) shows the ac susceptibility measurement setup with the Helmholtz excitation coil and cryostat sitting on an alignment frame. The SQUID chip is placed inside a liquid nitrogen cryostat made from non-magnetic materials (fiber-glass nitrogen bath and polyvinyle chloride (PVC) vacuum shell). The cold part of the cryostat is separated from the room temperature environment by a 250 *μ*m thick sapphire window. To avoid physical contact, the SQUID gradiometer is maintained about 1 mm from the top surface of the window. Such a small separation increases the coupling between the sample and the gradiometer pick-up loops as the magnetic field rapidly decays from the source. A Helmholtz coil of 30 cm in diameter surrounds the SQUID and the sample and provides ac excitation field amplitudes up to 40 *μ*T in the frequency range of 1 Hz–10 kHz. To minimize the coupling between the excitation field and the SQUID, the Helmholtz coil is aligned in the plane of the SQUID and parallel to the baseline of the gradiometer pick-up loops. A Fluke AWG-220 waveform generator drives the Helmholtz coils to produce the excitation field. The SQUID signal is read out by Magnicon^®^ SEL-1 electronics in the flux locked loop mode. A Stanford research systems^®^ SR-830 lock-in amplifier is used to obtain the real and imaginary components of the magnetic susceptibility from the SQUID gradiometer versus the excitation frequency in the range of 1 Hz to 10 kHz.

### D. Magnetic nanoparticles

Magnetite (Fe_3_O_4_) multicore-shell nanoparticles with a median particle diameter of 100 nm suspended in PBS solution were used in our experiments. These particles are available in colloidal solutions of 10 mg/ml concentration and have $6.0\times 10^{12}$ MNPs per mL according to the company's technical data sheet (Micromod Partikeltechnologie GmbH, Rostock, Germany, https://www.micromod.de/pdf/aktuell/10–19-102_tds_en.pdf). The magnetic cores in the multi-core particles have a median size of 15 nm with a starch shell, which is capped with the streptavidin surface for binding of biotinylated molecules. The particles were functionalized by adding biotin conjugated oligonucleotide probes to the surface of the MNPs (Zardán Gómez de la Torre *et al.*, 2011). Brownian relaxation dominates dynamics for the used magnetic multi-core particle system.

### E. Target DNA molecule recognition and amplification

In order to detect the target DNA molecules, we use padlock probe recognition (Nilsson *et al.*, 1994; Zardán Gómez de la Torre *et al.*, 2011) followed by RCA (Banér *et al.*, 1998; Zardán Gómez de la Torre *et al.*, 2011). A linear padlock probe molecule with matching motifs to the two ends of the target DNA sequence is hybridized to the sequence through ligation and forms a DNA circle. These circularized padlock probes are amplified upon addition of phi 29 DNA polymerase to create long DNA molecules that collapse into DNA coils. The size of the coils depends on the amplification time; in our experiments, the amplification time is 60 min, resulting in a coil diameter of about 1 *μ*m. The oligonucleotide tagged magnetic nanoparticles are then incorporated into the large DNA coils by base pair hybridization. As a result, the conjugated fMNPs will have a much larger hydrodynamic volume compared to the free MNPs. Complete details of the method for the target recognition, ligation, and amplification are given in Zardán Gómez de la Torre *et al.*, 2011.

All different dilutions of RCA coil samples contained the exactly same initial mass concentration of fMNPs (50 *μ*g/ml) for hybridization. To minimize pipetting errors, the samples were prepared at relatively large volumes of 400 *μ*l compared to the 3 *μ*l sample volumes which were measured in the microchannel. During the hybridization process, the volume and concentration of both the MNPs and RCA coils were kept small and high, respectively, in order to increase the likelihood of the fMNP and RCA coil conjugation. Samples were then diluted to the desired final volume and concentration by simply adding a hybridization buffer containing 40 mM Tris-HCl (pH 8), 40 mM EDTA, 0.2% Tween-20, and 1 M NaCl. In all the samples and experiments, synthetic target bioanalytes were used and ethics approval was therefore not required.

### F. Brownian relaxation

The ac susceptibility measurement is based on the detection of the dynamic magnetic properties of suspended magnetic particles (Fannin *et al.*, 1988). The effective relaxation for the liquid suspended MNPs is a combination of Néel and Brownian relaxations. However, for multi-core particles with a total median size of 100 nm and magnetic cores with a median size of 15 nm, the magnetic relaxation is dominated by Brownian relaxation in the frequency window of our measurements (1 Hz–10 kHz). The Brownian relaxation time (*τ_B_*) and frequency (*f_B_*) are given by

$$\tau_{B}=\frac{1}{2\pi f_{B}}=\frac{3\eta V_{H}}{kT},$$ (1)

where η is the dynamic viscosity of the liquid, *k* is Boltzmann's constant, *T* is the absolute temperature, and $V_{H}$ is the hydrodynamic volume of the MNP. The MNPs conjugated to the RCA coils will have much larger $V_{H}$ as compared to free MNPs. Therefore, their relaxation time is much longer according to Eq. (1). To measure the relaxation time, we apply an ac magnetic field and measure magnetic ac susceptibility (real and imaginary components) of the MNPs as a function of excitation frequency. The imaginary part of the ac susceptibility has a peak at the effective relaxation frequency $f_{B}=1/{2\pi \tau }_{B}$. Since the change in $V_{H}$ is large, one can use the extinction of the imaginary part of the susceptibility of free MNPs upon conjugation with RCA coils as the primary detection signal.
